# KvarQ: targeted and direct variant calling from fastq reads of bacterial genomes

**DOI:** 10.1186/1471-2164-15-881

**Published:** 2014-10-09

**Authors:** Andreas Steiner, David Stucki, Mireia Coscolla, Sonia Borrell, Sebastien Gagneux

**Affiliations:** Swiss Tropical and Public Health Institute, Socinstrasse 57, Basel, 4051 Switzerland; University of Basel, Basel, Switzerland

**Keywords:** Whole genome sequencing, FastQ, Single nucleotide polymorphisms, In-silico SNP-typing, Mycobacterium tuberculosis

## Abstract

**Background:**

High-throughput DNA sequencing produces vast amounts of data, with millions of short reads that usually have to be mapped to a reference genome or newly assembled. Both reference-based mapping and *de novo* assembly are computationally intensive, generating large intermediary data files, and thus require bioinformatics skills that are often lacking in the laboratories producing the data. Moreover, many research and practical applications in microbiology require only a small fraction of the whole genome data.

**Results:**

We developed KvarQ, a new tool that directly scans fastq files of bacterial genome sequences for known variants, such as single nucleotide polymorphisms (SNP), bypassing the need of mapping all sequencing reads to a reference genome and *de novo* assembly. Instead, KvarQ loads “testsuites” that define specific SNPs or short regions of interest in a reference genome, and directly synthesizes the relevant results based on the occurrence of these markers in the fastq files. KvarQ has a versatile command line interface and a graphical user interface. KvarQ currently ships with two “testsuites” for *Mycobacterium tuberculosis*, but new “testsuites” for other organisms can easily be created and distributed. In this article, we demonstrate how KvarQ can be used to successfully detect all main drug resistance mutations and phylogenetic markers in 880 bacterial whole genome sequences. The average scanning time per genome sequence was two minutes. The variant calls of a subset of these genomes were validated with a standard bioinformatics pipeline and revealed >99% congruency.

**Conclusion:**

KvarQ is a user-friendly tool that directly extracts relevant information from fastq files. This enables researchers and laboratory technicians with limited bioinformatics expertise to scan and analyze raw sequencing data in a matter of minutes. KvarQ is open-source, and pre-compiled packages with a graphical user interface are available at http://www.swisstph.ch/kvarq.

**Electronic supplementary material:**

The online version of this article (doi:10.1186/1471-2164-15-881) contains supplementary material, which is available to authorized users.

## Background

Large-scale whole-genome sequencing (WGS) is revolutionizing microbiology and public health [1–3]. Thanks to the latest technological advances, bacterial genomes can now be sequenced in less than 48 hours for less than €100 per genome. New benchtop devices are providing WGS capability to routine microbiological laboratories, and WGS is now becoming an important part of clinical microbiology and molecular epidemiology. This emerging field of “genomic epidemiology” [4–11] already comprises numerous studies that have evaluated the potential of WGS to trace the transmission of pathogens such as *Clostridium difficile*, *Pseudomonas aeruginosa*, *Klebsiella pneumonia*, *Neisseria meningitidis*, *Staphylococcus aureus* and *Mycobacterium tuberculosis* [12–21]. Moreover, WGS is playing an increasing role in molecular drug susceptibility testing (DST) and the identification of drug resistance mutations [22–28]. It is expected that WGS will eventually replace all other bacterial genotyping methods [29].

In contrast to the increasing availability of WGS platforms, the capability to analyse WGS data is lagging behind, partially because of the lack of rapid and user-friendly analysis tools. Extracting single nucleotide polymorphisms (SNPs), the most widely studied form of genetic variation, from WGS data requires substantial bioinformatics expertise, as well as dedicated bioinformatic analysis pipelines often based on customized scripts. Moreover, this standard approach of analysing WGS data is time-consuming and computationally expensive [30], and as a consequence, biological questions can often not be addressed directly by the people generating the data. Furthermore, large amounts of data are produced when analyzing complete genomes, although often only a small proportion of these data are relevant for the study question; this is particularly true when screening for drug resistance determinants. Identifying individual SNPs of interest usually requires mapping all sequencing reads in a fastq file to a given reference genome, despite the fact that only a few nucleotide positions might be relevant. Tools have been developed to speed up the extraction of relevant information. These include *in silico* multi-locus sequence typing (MLST) from draft genomes or contigs, but all these methods rely on previous assembly [31–33]. One of these tools, SRST, facilitates the process by accepting fastq files for a given typing scheme. SRST uses BWA and SAMtools and is highly configurable, but is aimed at the bioinformatically skilled users and lacks a graphical interface. However, a graphical interface is crucial for making these kinds of genome analyses accessible to a broader group of microbiologists, including the ones primarily interested in routine clinical applications. Consequently, there is a need for software able to extract allelic information directly from the raw sequencing reads without the need of previous mapping or *de novo* assembly.

Here we present KvarQ, a software that enables rapid screening of short sequence reads for mutations at multiple nucleotide positions of interest. KvarQ uses as input a fastq file, and generates the output in form of a text file in JavaScript Object Notation (json) format. Using a complete bacterial genome sequence as a reference, KvarQ can interrogate any single nucleotide position or short DNA sequence of interest for known or unknown mutations, respectively.

KvarQ is universally applicable to any short read dataset and corresponding reference sequence, and is highly adaptable with respect to target polymorphisms. Clinical microbiological laboratories will benefit from KvarQ’s short analysis time, while laboratories in resource-limited settings can get access to genome analysis despite the lack of advanced computational infrastructure and/or appropriately skilled staff.

Even advanced users will save time by avoiding the need for formal mapping or assembly to answer specific questions, particularly when studying large numbers of genomes.

KvarQ is available for Linux, OS X and Windows, and runs on any portable or desktop system. A command line interface and a simple graphical user interface are available. We aimed at a self-explaining application with short run times (<20 minutes on a standard workstation for a fastq file of 1000 MB), while keeping the program flexible enough to be used with other organisms. KvarQ can be downloaded (source code as well as precompiled packages) from http://www.swisstph.ch/kvarq.

## Implementation

### Overview

KvarQ analyzes a fastq file and detects the allelic state of known polymorphisms, and compiles all the relevant information about the genome sequence. In contrast to current genetic analysis software [31, 33], KvarQ extracts the relevant information directly from the sequencing reads, without the need to map every read to a reference genome. The software can be used via the command line or the graphical user interface, and is split into one part that scans the fastq files and saves the results to a json file, and a second part that extracts and illustrates the information contained in the json file. Although results reported in this article mainly use sequencing data from the *Mycobacterium tuberculosis* complex (MTBC), the software can be used with any short read sequencing data from any organism. Currently, KvarQ is optimized for haploid organisms, but could be extended to analyze diploid data sets. In principle, KvarQ could be used to scan through very large datasets, such as from human genomes, although the scan time would increase linearly with data size.

### Algorithm and parameters

**Figure 1:**
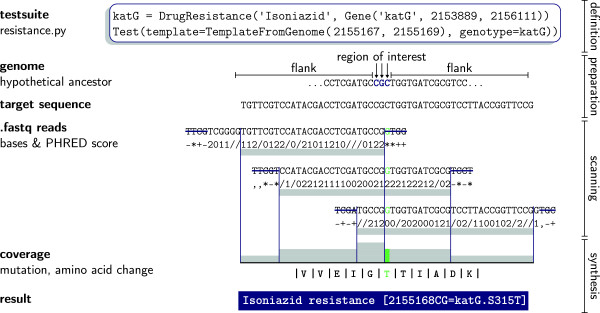
**Simplified overview of scanning process and preparation.** “Testsuites” are python source files that define the SNPs of interest (or in this case a 3 base pair long region) as well as other relevant genetic information (in this case the *katG* gene in which mutations can confer isoniazid resistance). This information is used to extract a “target sequence” from a reference MTBC genome: on both sides, additional bases (“flanks”) are concatenated to avoid border effects within the sequence of interest. During the scanning process, every read is trimmed depending on its PHRED score (in this case, a quality cutoff of Q = 13 was defined which corresponds to the ASCII character “/”). After the scanning, the part of the reads that matched the target sequence and exceeded the minimum quality score (represented with gray bars) are assembled to a “coverage” that indicates the overall coverage depth as well as all detected mutations (green). In a further step, additional information is generated from this coverage (such as the resulting amino acid sequence) and finally a short “result” string is generated that summarizes the result of the scanning process.

The following paragraphs describe the information flow inside KvarQ from the fastq files to the final results that are displayed to the user (Figure 1).

**Figure 2:**
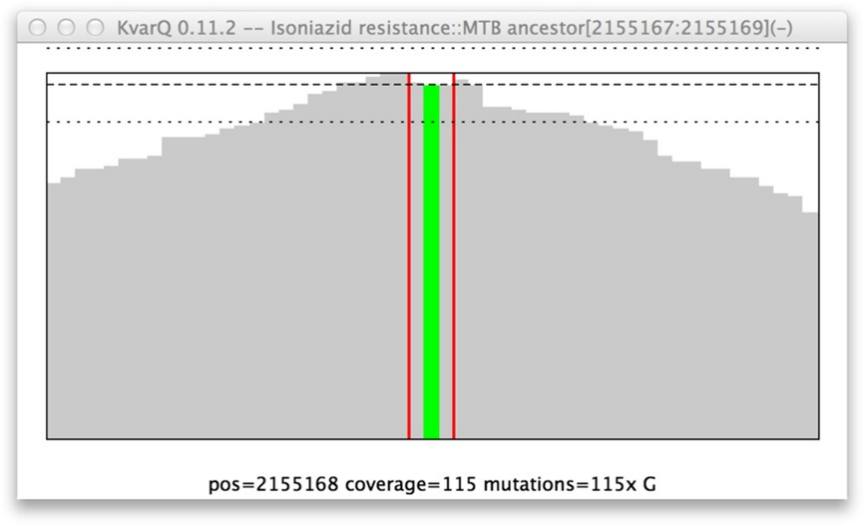
**Interactive inspection of “coverage”.** This screenshot shows the coverage over a short region of interest (3 base pairs within vertical red lines, corresponding to codon 315 in *katG*) and the surrounding flanking sequence. Note the mutation in the middle of the three base pair long sequence (in green, text description at bottom of window). The decrease of coverage on both sides is due to the minimum overlap that is set to a value equal to the length of the two flanking regions.

Before the scanning process, target sequences are generated from known mutations or regions of interest. KvarQ uses a modular approach, where different “testsuites” (realized as python modules) define known single nucleotide polymorphisms (SNPs) or small regions of interest that harbour potential mutations (such as the Rifampicin Resistance-Determining Region, RRDR, in MTBC [34]). The target genomic sequence is extracted from a reference genome and flanked on both sides with additional bases. These flanking regions increase the length of the sequence and thereby increase the coverage over the region of interest (see Figure 2). For each of these “target sequences”, the corresponding base-sequence on the complementary strand is also generated.

The actual scanning algorithm is implemented in C and splits the fastq file into small parts that are distributed to multiple threads and scanned simultaneously. First, every read is trimmed by a user-specified minimum quality score. The trimmed part is then shifted along each of the target sequences and every match (not exceeding a specified number of single nucleotide differences) is recorded in the “hit-list” if a minimum overlap is warranted. Currently, no indexing/caching is used for comparison of the reads with the target sequences. This works fast enough for the short target sequences used in our analysis. The scanning process continues until the whole file is scanned or a specified minimum coverage is reached.

Next, the data gathered from the fastq files is translated into intermediary data structures (“coverages”). These data structures combine the reads from the original as well as the complementary strand, position them within the genome, and summarize non-matching bases in reads (these can be true mutations in the genome or sequencing errors).

Finally, the testsuites determine the results based on the information conveyed by the intermediary data structures created in the last step. Currently, mutations are extracted by using a simple threshold that separates them from sequencing errors – this is possible because KvarQ neglects bases that do not satisfy the minimum read quality requirements set by the user. How this mutation information is processed further varies depending on the various testsuites. For example, the phylogenetic testsuite we applied for MTBC uses three phylogenetically informative SNPs for every lineage and imposes logical constraints on *sub*lineages. For example, any genome belonging to the MTBC “Beijing” sublineage must also have the SNPs characteristic of “Lineage 2”. The resistance testsuite checks for every mutation whether a change in amino acid sequence follows the nucleotide change (non-synonymous mutations) and only reports those to the user. If the results have a low confidence due to low coverage of the sequences, this is also added as a remark to the result output.

### Data format and analysis

KvarQ uses fastq files with Solexa, Sanger and different Illumina quality scores as input files. The quality format is determined by a heuristic search. Only one input file per genome sequence is accepted, and paired-end files are automatically included. The output is structured data in json format, a human readable file that contains information about the scanning process (parameters used, fastq file size, statistics about read number, length and quality, and scan time), the final results, and the intermediary data structures that were used to calculate these results. The information contained in the json file can be used to re-calculate the results without the need to re-scan the fastq file, because most of the relevant information is contained in the intermediary data structures that are saved along with the results in the json file.

**Figure 3:**
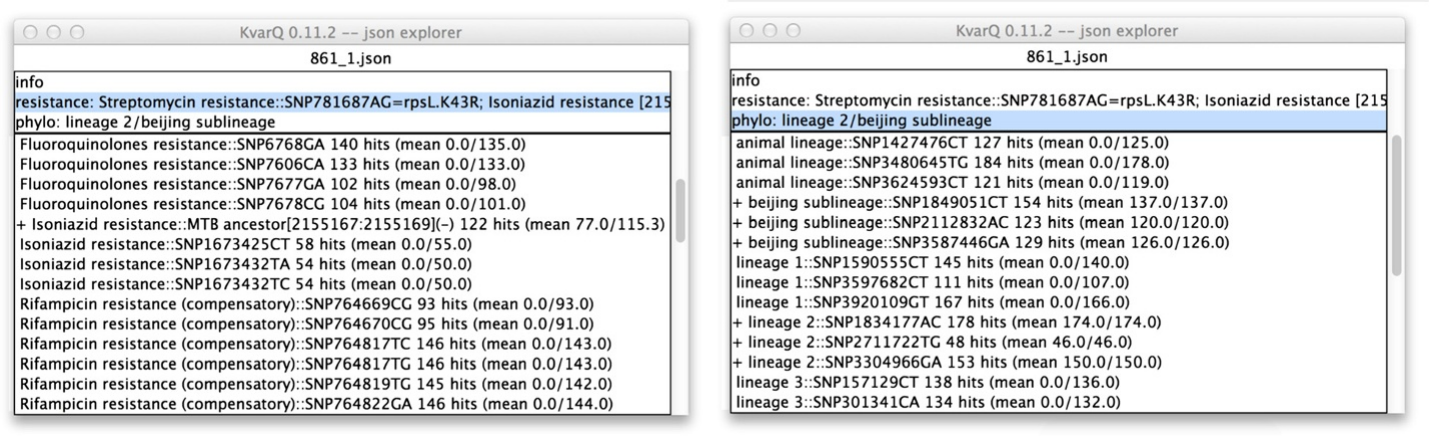
**Interactive inspection of json file.** The upper pane of each window in these screenshots shows the main categories of data contained in the json file. In the left window, the drug resistance section is selected and the lower pane shows details about all target sequences in this testsuite (the “+” in front of the “Isoniazide resistance” indicates a non-synonymous mutation). In the right window, the phylogenetic section is selected, showing that all SNPs for “lineage 2” and “beijing sublineage” were found.

To facilitate the extraction of relevant results, KvarQ provides data analysis tools to inspect the data interactively from the command line or with a hierarchical menu-driven graphical user interface. The “json explorer” shows the summarized test results for every testsuite (KvarQ’s main goal is to be user-friendly) as well as detailed information about the coverage of every target sequence that was used by the different testsuites (Figures 2 and 3). The interactive exploration of this wealth of information is intended for the advanced operator to get, for example, a better impression of the usefulness of newly designed target sequences or the fastq quality. Additionally, the “json explorer” displays overall number of reads in a fastq file and the length of the reads.

## Results

We validated KvarQ with 880 fastq files of predominantly *Mycobacterium tuberculosis* complex (MTBC) isolates, for which we interrogated 206 genomic positions where polymorphisms have been previously described (Additional files 1 and 2).

In MTBC, single nucleotide mutations are reliable drug resistance markers, as most phenotypic drug resistance is conferred by single amino acid changes [34–36]. Hence, molecular DST is becoming increasingly widespread in the diagnosis of TB, and various genotyping platforms make use of these markers. Moreover, SNPs are powerful phylogenetic markers in MTBC, because similar to other genetically monomorphic bacteria, MTBC exhibits limited horizontal gene exchange and as a consequence, SNP homoplasies are rare [37, 38]. Hence, SNPs can be used to study the evolutionary history of MTBC, as well as to classify clinical strains associated with variable degrees of virulence or different clinical outcomes [39, 40]. MTBC comprises several phylogenetic lineages, and SNPs represent ideal markers to classify clinical MTBC isolates into these lineages [41, 42].

In this study, we included 27 SNPs as phylogenetic markers defining MTBC lineages and sublineages, as well as 32 single nucleotide mutations and three genomic regions (63 base pairs (bp) in *gyrA*, 81 bp in *rpoB* and 3 bp in *katG*) associated with drug resistance (Additional file 1).

**Figure 4:**
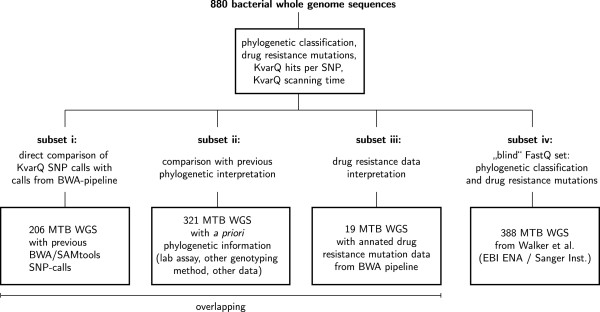
**FastQ dataset used to validate KvarQ.** A total of 880 whole genome sequences in FastQ format from various sources were used in this study. All 880 genome sequences were scanned for phylogenetic classification and drug resistance mutation identification. Different, overlapping subsets were used to *i)* compare SNP calls obtained with KvarQ with SNP calls of our standard SNP-calling pipeline based on BWA and SAMtools for 206 isolates, *ii)* compare KvarQ phylogenetic classification of 321 MTBC isolates with previous phylogenetic information, *iii)* compare KvarQ drug resistance mutations with previously identified drug resistance associated mutations in 19 MTBC isolates, and *iv)* obtain additional information, i.e. phylogenetic classification and drug resistance mutations, from a “blind” set of 388 genome sequences from a recent study [21]. More information can be found in Additional file 2.

We tested KvarQ with a set of “in-house” generated whole genome sequences of clinical MTBC isolates and additionally downloaded sequences from public sequence read archives (Additional file 2). Different, overlapping subsets of fastq files were used for *i)* the comparison of SNP-calls with a standard BWA-based mapping pipeline (N = 206), *ii)* detecting drug resistance mutations (N = 19) and *iii)* comparison of KvarQ phylogenetic classification with laboratory based classification (N = 321) – see Figure 4. In addition to these well-characterized genome subsets, we used a previously published “blind” subset of 388 fastq files downloaded from a public source to extract phylogenetic information and drug resistance mutations, which were not reported in the original publication [21].

### KvarQ scanning times and overall performance

**Figure 5:**
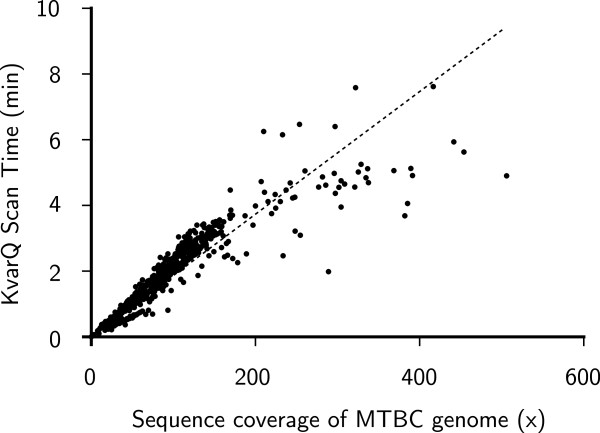
**KvarQ scanning time for all 206 polymorphic positions with respect to average sequence coverage of the MTBC genome per fastq file.** Each dot represents one isolate (i.e. fastq file). Scanning time was found to be 112 seconds/100× coverage. Eleven isolates of 880 were excluded due to variable read lengths (difficult calculation of total base output). Three additional isolates were excluded because of file sizes larger than 10 GB.

First, we applied KvarQ to all 880 fastq files and calculated scanning times for the set of SNPs described. The scanning times were found correlated with the average genome coverage (or the total base output) in the fastq file. A genome sequence file with 100-fold coverage of the MTBC genome required approximately 2 minutes of KvarQ scanning time, and increased linearly with sequence coverage (Figure 5).

**Figure 6:**
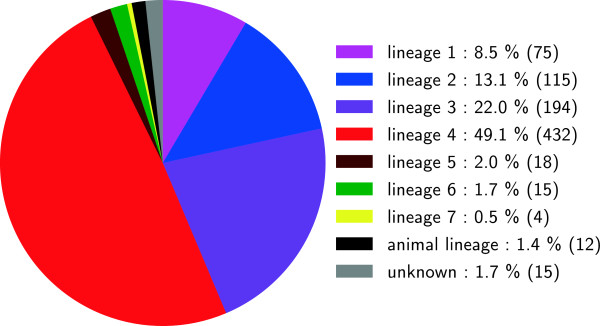
**Phylogenetic classification of all 880 isolates used in this study.** This figure shows the distribution of phylogenetic markers in all of the scanned genomes. For 865 isolates, an MTBC lineage-specific SNP was found. In 15 isolates, no MTBC lineage-specific SNPs were found, either because isolates were non-MTBC (10 were known to be non-MTBC), or because coverage was low.

To assess overall performance, we looked at phylogenetic classifications in all 880 fastq files. These files included whole genome sequences generated on different sequencing devices using different laboratory procedures (i.e. library preparations), and of unknown quality (Additional file 2). Successful phylogenetic classification was obtained for 865/880 fastq files (98.3%) (Figure 6). The remaining 15 included the chimpanzee bacillus [43], *Mycobacterium canettii* and eight confirmed non-MTBC isolates, for all of which no MTBC lineage classification would be expected. For five other strains, no MTBC lineage-specific SNP was detected, which can be due to low coverage or because isolates were not MTBC.

Furthermore, we compared the number of KvarQ hits per position with overall base output in the fastq file (data not shown). The correlation varied strongly between positions on the genomes and between fastq files, which indicates that the number of hits per SNP depends on the SNP queried (i.e. the particular genomic region being interrogated) and the quality of the base calls in the fastq file.

### Comparison of SNP-calls with BWA calls

A subset of 206 FastQ files was used to directly compare SNP-calls obtained with KvarQ with SNP-calls generated with our standard pipeline using BWA, SAMtools, BCFtools and custom scripts for filtering [43]. For each of the 206 positions (single nucleotide positions plus regions of interest), the BWA list of variants in the corresponding genome was interrogated for presence of an alternative allele compared to the reference genome (reconstructed hypothetical ancestor [38, 41]). With this, a direct comparison of all 206 positions in 206 strains was performed, resulting in 42,436 data points (Table 1).

**Table 1:** **Comparison of SNP-calls at 206 polymorphic positions in 206 MTBC genome sequence fastq files**

	SNP in BWA mapping pipeline					
	Yes		No		Total	
	N	%	N	%	N	%
SNP in KvarQ	782	99.24	33	0.08	815	1.92
no SNP in KvarQ	6	0.76	41615	99.92	41621	98.08
Total	788	100	41648	100	42436	100

A total of 782 mutations were called by both KvarQ and the BWA-pipeline (1.8% of all data points). This corresponds to a sensitivity of 99.2% in mutation calling of KvarQ (782/788) taking the BWA pipeline as the gold standard. Specificity was found to be 99.9% (41,615/41,648 positions).

Thirty-three mutations (0.08% of all data points) in 20 fastq files were called by KvarQ, but no mutation was found in the corresponding BWA SNP-list. Upon manual inspection, we found a successful SNP-call in the SAMtools *pileup*-list for each of the 33 mutations, but these mutations were filtered out in the subsequent heuristic filtering step including the SAMtools varFilter command for unknown reasons and despite good quality and coverage.

We found three fastq files where KvarQ missed a single SNP and one file where KvarQ missed 3 SNPs despite six calls in the BWA-pipeline. The BWA read depths for these six calls (0.01% of all data points) were 5, 6, 21, 38, 64 and 77. Corresponding KvarQ number of hits were 0, 1, 2, 3, 4 and 5, respectively. For strain MTB_erdman_SRR017677, when the KvarQ quality cutoff was removed (all reads considered), the number of hits increased to 57, 84 and 120, respectively. Subsequent FastQC analysis (http://www.bioinformatics.babraham.ac.uk/projects/fastqc/) showed poor fastq quality over the whole read length (data not shown).

### Validation of phylogenetic classification

A subset of 321 isolates was used to compare the phylogenetic classification by KvarQ with data obtained previously by various genotyping methods (molecular assays in our laboratory [40]), data obtained from the collaborating laboratory that provided DNA for WGS, or metadata from the sequence read archive in the case of downloaded files. The comparison included seven main phylogenetic lineages [41], the animal-associated MTBC clade (*M. bovis*/*M. caprae*), the Beijing sublineage of Lineage 2, and 10 non-MTBC isolates. In 309/321 (96.3%) isolates, KvarQ detected MTBC lineage-specific SNPs that were in agreement with the previous classifications (Additional file 2). The remaining 12 genome sequences for which no MTBC lineage-specific SNP was found included eight non-MTBC, the chimpanzee bacillus and *M. canettii*, for which no MTBC-lineage specific SNP was expected, and two isolates (0.6%) with low KvarQ coverage and therefore no lineage-call.

### Drug resistance associated mutations

KvarQ was applied to a subset of 19 strains harbouring known mutations associated with drug resistance to validate the interpretation (annotation) of the mutations. The 19 strains overlapped with the 206 strains of the direct comparison described above, but the resistance-associated mutations were identified with separate scripts from the annotated SNP-list in the BWA pipeline. The 179 drug resistance associated genomic positions were interrogated with KvarQ, including the 81 bp Rifampicin Resistance Determining Region (RRDR) of *rpoB*, the 63 bp Quinolone Resistance Determining Region of *gyrA* (QRDR) and 3 bp in the codon 315 of *katG* (Additional file 1).

Among the 19 strains, 16 (84%) had perfectly congruent drug resistance mutations identified by KvarQ when compared to the BWA pipeline. For one strain (GQ1580), one mutation in *rpoC* was not found by KvarQ. However, this mutation was not in the list of target SNPs (Additional file 1). For one isolate, MTB_KZN_605, an additional mutation in *gyrA* was found with KvarQ, and was not found with the BWA pipeline due to low read depth and the presence of an alternative allele. For another isolate, MTB_russia_ERR015616_1, six additional mutations in five genes were found by KvarQ. All mutations in these two isolates were traced back to the filtering problem discussed above, and were found in the *pileup* file.

**Figure 7:**
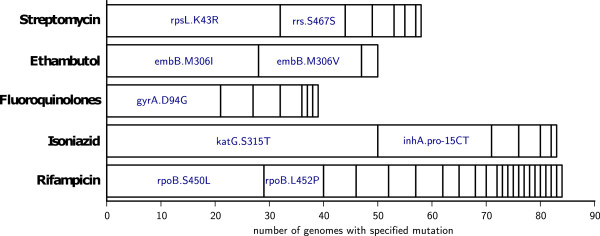
**MTBC drug resistance associated mutations found in all 880 isolates.** This figure shows all 314 mutations that were found with KvarQ in 139 isolates with at least one mutation in any of the drug resistance associated genes that were analysed (see Additional file 1). Only mutations that were found in at least ten isolates are labeled.

In total, KvarQ detected 314 drug resistance associated mutations in 139/880 files (15.8%) (Figure 7).

### “Blind” set of fastq files from a public repository

**Figure 8:**
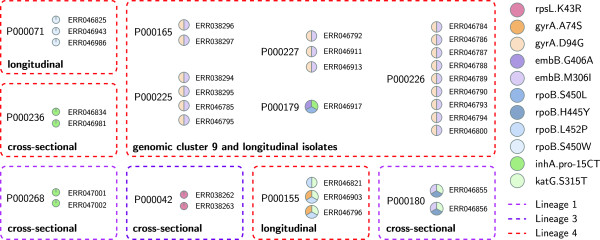
**KvarQ identified 62 drug resistance associated mutations in 33 of 388 patient isolates in a “blind” set of genome sequences from Walker et al.**[21]**.** Each circle represents one isolate (Patient number given as P-number, isolate given as ERR-number). Only isolates with drug resistance associated mutation identified by KvarQ are shown. The 62 individual drug resistance mutations are shown as colors of pies. Phylogenetic lineages were obtained with KvarQ and are shown as colored and dashed boxes. Group definitions were obtained from the original publication [21]: cross-sectional isolates were paired pulmonary and extrapulmonary isolates from the same patient, longitudinal isolates were paired isolates from the same patient separated by at least 6 months, and genomic cluster 9 was a large MIRU-VNTR defined cluster of transmission in the community. Paired isolates (longitudinal and cross-sectional) as well as isolates of cluster 9 were found to harbour the same mutations, except for P000179, where three otherwise not detected mutations were found by KvarQ. For patient P000155, an additionally acquired mutation was found in two of three isolates. Two of the 390 whole genome sequences in the original publication were not found for download.

**Figure 9:**
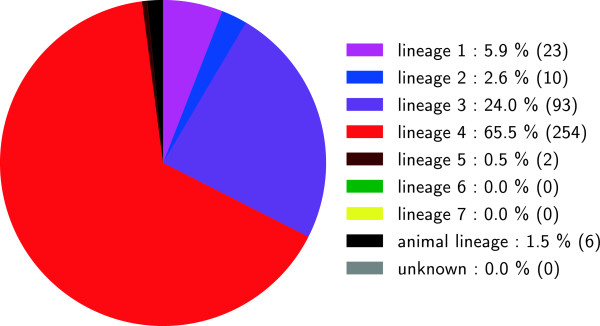
**Main MTBC phylogenetic lineage classification of the fastq set of Walker et al.** [21]**, consisting of 388 isolates.** Successful lineage assignment was obtained for 388/388 (100%) isolates. Figure includes all clustered isolates as well as longitudinal, cross-sectional and household isolates.

Finally, a “blind” set of whole genome sequences was used to illustrate the added value provided by KvarQ. We downloaded available sequences of a recent publication that used whole genome sequencing for molecular epidemiology of MTBC transmission [21]. This publication did not report drug resistance data or phylogenetic data. We therefore analyzed all available sequences for phylogenetic SNPs and drug resistance mutations. The original sample set consisted of 390 isolates, but two fastq files were not found for download (http://www.sanger.ac.uk, ERR192249 and ERR192250). Results for 388 isolates were obtained in less than 12 hours. A total of 62 drug resistance mutations were identified in 33 patient isolates from 11 patients (Figure 8). All paired isolates (cross-sectional: paired pulmonary and extrapulmonary isolates from the same patients; longitudinal: paired isolates from the same patient separated by at least 6 months; shown as smaller dotted boxes in Figure 8) were found to harbour identical drug resistance associated mutations, except for one patient (P000155), where an additional mutation was found in two of three isolates. Isolates of one community transmission cluster (defined by MIRU-VNTR), cluster 9, were found to harbour drug resistance associated mutations (large box in Figure 8). Within cluster 9, all patient isolates harboured the same drug resistance mutations, except for one patient (P000179) with three otherwise unidentified mutations. Among the 388 isolates, we found a predominance of Lineage 4 (i.e. the Euro-American lineage) in the sample set (65.5%), followed by Lineage 3 (24.0%) and Lineage 1 (5.9%) (Figure 9).

## Discussion

In this article, we propose a new approach to *in silico* SNP-typing from whole-genome sequencing data. KvarQ scans fastq short sequence reads directly for known polymorphisms instead of mapping or assembling the complete genome. This results in a massively reduced computation time required compared to established WGS analysis tools. At the same time, KvarQ offers a high degree of flexibility and is also user-friendly. This was achieved by developing an extended command-line interface together with pre-compiled packages with a graphical user interface.

KvarQ was designed for bacterial genomes, but is applicable to any fastq sequence data. The default parameters work best for the different Illumina machines, but can easily be adapted to data from other manufacturers. For example, the Roche 454 reads in our testset were scanned with a lower quality cutoff. Due to the lack of relevant data from MTBC isolates, we were not able to test reads from Pacific Bioscience devices.

Both the reference sequence and the interrogated polymorphisms can be changed or extended. Changing the SNPs of interest can be achieved by modifying the templates in the testsuites, which are lists of nucleotide positions screened by KvarQ. An extended documentation including instructions for changing the target polymorphisms is distributed with the software and available online (http://www.swisstph.ch/kvarq). Scanning parameters are adjusted by invoking a number of options in the command-line interface or via the settings dialog in the graphical user interface.

Using our test set of 880 fastq files, we obtained KvarQ running times of less than 2 minutes on a high-performance computer (average running time 110 seconds) and less than 20 minutes on a standard desktop computer (Figure 5). This important decrease in time for analysis (when compared to the conventional pipeline in which the whole genome sequence is first reconstructed) is crucial for the efficient treatment of data as WGS becomes more widely available. The overall performance of KvarQ was high, as reflected by 98.3% of genome sequences successfully assigned to a phylogenetic MTBC lineage (Figure 6). By including phylogenetically redundant SNPs, the sensitivity of classification was increased. Specifically, classifications were obtained if 2 of 3 redundant SNPs were found mutant. The 96.3% congruence of lineage-assignment of KvarQ with *a priori* lineage classification illustrates how WGS combined with a quick tool such as KvarQ has the potential to replace previous genotyping methods.

When comparing KvarQ SNP-calls with calls from our mapping pipeline using BWA and SAMtools, we found a high sensitivity and specificity of 99.2% and 99.9%, respectively. The three mutations that were called by the BWA pipeline but not by KvarQ were resolved when lowering the quality cutoff parameter. FastQC (http://www.bioinformatics.babraham.ac.uk/projects/fastqc/) confirmed the low quality of the reads in these files.

Drug resistance mutations obtained with KvarQ and BWA in the 19 compared files were nearly identical, but showed one limitation. Mutations conferring resistance to pyrazinamide are found in any part of *pncA*, which is 561 bp long [44]. KvarQ was designed to scan short stretches of DNA, and we therefore excluded mutations in *pncA* to avoid an increase in scanning time.

In the total set of 880 genomes, we found many additional drug resistance associated mutations (Figure 7) that could not be validated due to the lack of laboratory DST data for many of the isolates and the complex genetic nature of drug resistance in MTBC [45].

Successful KvarQ calls depended on the selection of suitable SNPs. Several SNPs had to be replaced in the testing phase. Most SNPs that were replaced occurred in repetitive regions, and these SNPs were also never called when using the BWA-pipeline. We therefore recommend including only SNPs that were previously found to be in core genome regions, in non-repetitive regions and where no deletions have been described.

With a “blind” set of genome sequences obtained from a recent publication [21], we were able to obtain lineage classifications and drug resistance calls, which had not been reported in the original publication, in less than one day. Using the clustering information of the publication, all isolates identified as drug-resistant by KvarQ and from the same patient (paired isolates) or the same patient cluster shared the same drug resistance mutations (except patient P000179, see Figure 8) and were assigned to the same phylogenetic lineage, highlighting the consistency in mutation calling. The fast generation of drug resistance and phylogenetic data illustrates the application of KvarQ for quick targeted scanning of large public datasets.

KvarQ was designed to interrogate positions with known mutations. However, as some regions (e.g. hot spot regions of drug resistance) can harbour several mutations in close proximity, we included the capability to scan regions of interest for the presence of any (new) mutation. These regions can be of any length, but the scanning time increases in square with sequence length. The drug resistance testsuite in the current version checks some short regions known to harbour many different drug resistance associated mutations and reports any new mutation that would result in a amino acid change of the associated gene. In future releases, KvarQ could be extended to scan for other genomic polymorphisms, such as small insertions and deletions (InDels) as well as large sequence deletions or duplications.

## Conclusion

In conclusion, KvarQ provides a user-friendly and highly flexible platform for rapid and targeted analysis of fastq files. KvarQ will help overcome the hurdles of whole genome sequence analysis in clinical microbiological laboratories and other settings where bioinformatics capacity is limited. The short running times, the user-friendly graphical interface and the high configurability at the command line level allows analysing hundreds of fastq files in a short time.

## Methods

### Informative SNPs for MTBC

We used known phylogenetic markers and drug resistance associated mutations of MTBC to validate and benchmark KvarQ when scanning genome sequences of clinical MTBC strains. Phylogenetically informative SNPs were selected from previous publications [40, 42]. For each of the seven main phylogenetic lineages of human-associated MTBC, plus the *Mycobacterium bovis*/*M. caprae* lineage, we included three redundant canonical SNPs as markers for the corresponding lineage. Previously published SNPs were complemented with SNPs obtained as described before [40] for cases where less than three SNPs per phylogenetic clade were available. The extraction of these additional SNPs was based on 172 genomes described by [41]. For Lineage 2, we additionally included known polymorphisms to discriminate the so-called “Beijing” lineage from non-Beijing Lineage 2 strains. An overview of the phylogenetically informative SNPs included in KvarQ is shown in Additional file 1. We included drug resistance-mutations obtained from the Tuberculosis Drug Resistance Database (TBDReaMDB) [46], and additionally compensatory mutations from [47]. High-confidence mutations for the most important anti-tuberculosis drugs were selected (isoniazid, rifampicin, ethambutol, streptomycin, fluoroquinolones and second-line injectable drugs). Pyrazinamide-resistance conferring mutations were excluded. Mutations in TBDReamDB are listed as codon changes, therefore we generated the corresponding nucleotide changes for inclusion in KvarQ. For rifampicin- and fluoroquinolone-resistance conferring mutations, we included the *rifampicin-resistance determining region* (RRDR) [34] and the *quinolone resistance determining region* (QRDR) [48], respectively, rather than specific positions. The codon 315 of *katG* was also treated as a region of three base pairs. All included mutations and regions associated with drug resistance mutations are listed in Additional file 1. All genomic positions given in this study refer to the genome of the strain H37Rv (NC000962.3/AL123456.3) [44, 49, 50].

### Short-read datasets

We used a test set of 880 bacterial whole genome sequences in fastq format to screen for mutations with KvarQ. This set represents a global and diverse collection of clinical isolates of MTBC, and includes drug-resistant strains from patients and from in vitro evolution experiments, as well as non-MTBC bacterial genome sequences. Additionally, the genome sequences were chosen to represent a technically diverse collection (Illumina HiSeq 2000, GAIIx and MiSeq). In addition to the genome sequences generated in-house, the test set contains fastq files downloaded from http://www.sanger.ac.uk, the European Nucleotide Archive (ENA; http://www.ebi.ac.uk/ena/) and the Sequence Read Archive (SRA; http://www.ncbi.nlm.nih.gov/sra). More information on the fastq files, including accession numbers, can be found in Additional file 2.

### Mapping data for comparison with KvarQ

To compare KvarQ SNP calls with SNP calls obtained from conventional mapping of fastq short sequencing reads to a reference, we used a previously published computational pipeline [43]. In brief, all short reads were mapped to a hypothetical reconstructed ancestor with BWA, reads were piled up with SAMtools and variants called with BCFtools [51]. Filtering for low quality calls was done with customized scripts. The 206 positions that were used in KvarQ for allele detection were extracted with a Python script and compared to the KvarQ calls in the corresponding sequence. Discrepant results were manually checked with Artemis [52].

### KvarQ parameters used

All KvarQ results were generated using default parameters, i.e. a quality cutoff of 13, a minimum read length of 25, a minimum overlap of 25, a minimum coverage of two reads for allele calls and a maximum of two errors per read. Parameters were adjusted for fastq files with low coverage or low quality values, and are reported in Additional file 2. In case of paired-end sequencing files, only one of two read files was used. Files were manually concatenated in case of low coverage (reported in Additional file 2).

### Hardware specification

KvarQ runs on any system that runs Python and has a POSIX threads implementation (this includes notably Windows, OS X and Linux). Analyses in this study were performed on a Red Hat Enterprise Linux Server release 5.9 (Tikanga) with four six-core AMD Opteron CPUs and 132 GB RAM, using 8 threads.

### Ethics statement

All the work in this study was performed using previously published data (see Additional file 2).

## Availability and requirements

**Project name:** KvarQ: Targeted and Direct Variant Calling from FastQ Reads of Bacterial Genomes

**Project home page:**http://www.swisstph.ch/kvarq

**Operating systems:** platform independent; requires Python 2.7, POSIX thread library; pre-compiled packages for OS X (10.6.8 and later) and Windows (7 and later)

**Programming Language:** Python/C

**Other requirements:** (none)

**Licence:** GNU GPL v3

## Electronic supplementary material

Additional file 1: **List of SNPs used as markers for phylogenetic classification of MTBC, and drug resistance markers used in this study.** (XLSX 13 KB)

Additional file 2: **List of fastq files used in this study including KvarQ results and additional information.** (XLSX 151 KB)
